# Soil biochar amendment affects the diversity of *nosZ* transcripts: Implications for N_2_O formation

**DOI:** 10.1038/s41598-017-03282-y

**Published:** 2017-06-13

**Authors:** Johannes Harter, Mohamed El-Hadidi, Daniel H. Huson, Andreas Kappler, Sebastian Behrens

**Affiliations:** 1 Geomicrobiology & Microbial Ecology, Center for Applied Geosciences, University of Tuebingen, 72074 Tuebingen, Germany; 2 Algorithms in Bioinformatics, Center for Bioinformatics, University of Tuebingen, 72074 Tuebingen, Germany; 3 Bioinformatics, Center for Informatics Science (CIS), Nile University, Giza, Egypt; 4 Department of Civil, Environmental, and Geo-Engineering, University of Minnesota, Minneapolis, 55455 MN USA; 5 BioTechnology Institute, University of Minnesota, Minneapolis, 55455 MN USA

## Abstract

Microbial nitrogen transformation processes such as denitrification represent major sources of the potent greenhouse gas nitrous oxide (N_2_O). Soil biochar amendment has been shown to significantly decrease N_2_O emissions in various soils. However, the effect of biochar on the structure and function of microbial communities that actively perform nitrogen redox transformations has not been studied in detail yet. To analyse the community composition of actively denitrifying and N_2_O-reducing microbial communities, we collected RNA samples at different time points from a soil microcosm experiment conducted under denitrifying conditions and performed Illumina amplicon sequencing targeting *nirK*, typical *nosZ* and atypical *nosZ* mRNA transcripts. Within 10 days, biochar significantly increased the diversity of *nirK* and typical *nosZ* transcripts and resulted in taxonomic shifts among the typical *nosZ*-expressing microbial community. Furthermore, biochar addition led to a significant increase in transcript production among microbial species that are specialized on direct N_2_O reduction from the environment. Our results point towards a potential coupling of biochar-induced N_2_O emission reduction and an increase in microbial N_2_O reduction activity among specific groups of typical and atypical N_2_O reducers. However, experiments with other soils and biochars will be required to verify the transferability of these findings to other soil-biochar systems.

## Introduction

Nitrous oxide (N_2_O) is a strong greenhouse gas that has, based on its radiative capacity, an almost 300-fold greater global warming potential than CO_2_^1^. Since 1750 its atmospheric concentration increased from approximately (estimates from ice cores) 270 to 324 ppb in 2011^2^. Microbial nitrogen transformation processes in soils, such as nitrification and denitrification, represent the world’s largest sources of atmospheric N_2_O^1, 3^. N_2_O emissions have been increasing mainly as a result of excessive application of nitrogen fertilization in agricultural systems. Due to the increasing demand for food and animal feed, N_2_O emissions will likely further increase in the future^3^.

Biochar, a carbonaceous solid manufactured for the deliberate purpose of applying it to soil^4^, has recently been suggested as a promising N_2_O mitigation tool^5–7^. Biochar is produced by thermal decomposition of organic biomass under low oxygen conditions and has gained a lot of attention among agronomists and soil scientists because of its soil quality-enhancing properties and its recalcitrance to biodegradation^4, 8, 9^. Biochar’s physicochemical properties vary strongly with the type of organic material used as feedstock and the charring conditions during production. However, many biochars are produced by slow pyrolysis of woody plant material at temperatures between 400 and 700 °C and these biochars often share common physicochemical properties such as a high carbon content, a neutral to alkaline pH, a large surface area, and a highly aromatic carbon structure^8, 10, 11^. For wood-derived but also for other biochars, it has been shown that soil biochar amendment can considerably improve soil quality (e.g. by impacting soil pH, water holding capacity, and nutrient retention), while simultaneously sequestering carbon from the atmosphere^12–15^. In addition, a recent meta-analysis which summarized the results of several laboratory and field studies demonstrated that the addition of wood-derived biochars to arable soils results in a significant decrease of N_2_O emissions^6, 16^.

In the field, highest N_2_O emissions are frequently observed under denitrifying conditions (oxygen limitation, high nitrate and organic carbon concentrations), which occur for example after heavy rain fall on fertilized soil^17, 18^. Denitrification is defined as the stepwise enzymatic reduction of nitrate (NO_3_^−^) to dinitrogen (N_2_), with nitrite (NO_2_^−^), nitric oxide (NO), and N_2_O occurring as obligate intermediates. Denitrification is a common functional trait in many facultative and strict anaerobic chemoorganotrophic bacteria and has been also detected in some archaea and fungi^19, 20^. The enzymes catalysing the reactions of the different reduction steps in denitrification are encoded by the functional genes *narG* and *napA* (nitrate reductases), *nirK* and *nirS* (nitrite reductases), *norB* (nitric oxide reductase), and *nosZ* (nitrous oxide reductase)^19, 21^. Because some denitrifiers lack a functional *nosZ* gene and nitrous oxide reductases are highly oxygen- and pH-sensitive, the last step of denitrification (N_2_O reduction to N_2_) is often impaired^22–24^. Since the microbial reduction of N_2_O to N_2_ via *nosZ*-encoded nitrous oxide reductases represents the only known sink of N_2_O^1^, this process directly controls the N_2_O/(N_2_O + N_2_) emission ratio and thus the quantity of nitrogen that is released as N_2_O. Until recently N_2_O reduction was thought to be a functional trait exclusively associated with ‘classical’ denitrifiers. These microorganisms usually contain, in addition to the *nosZ* gene, also the functional genes that encode the enzymes catalysing the other steps towards complete denitrification (*narG*/*napA*, *nirK*/*nirS*, and *norB*). Most of the ‘classical’ denitrifiers are affiliated with the phylum Proteobacteria^19, 25^. However, recent studies revealed that the ability to perform N_2_O reduction is not restricted to these functional groups of microorganisms, but instead is also very common among microbes that belong to other phyla such as Actinobacteria, Bacteroidetes, Firmicutes and Chloroflexi^26, 27^. The majority of these microorganisms carry a fully functional but atypical form of the *nosZ* gene and about half of them do not contain a nitrite reductase gene^27–29^.

Although it has been confirmed in many studies that soil biochar amendment holds the potential to act as promising N_2_O mitigation strategy the underlying processes causing a decrease in N_2_O emissions are still not fully understood. Because N_2_O is formed during microbial nitrogen transformation reactions such as nitrification and especially denitrification, several studies aimed to link N_2_O emission suppression to changes in the structure and activity of microbial communities involved in nitrogen cycling^30–35^. It is known from 16S rRNA gene-based molecular fingerprinting and sequencing studies that the addition of biochar can significantly affect the microbial diversity in soil. Several studies reported a biochar-induced increase in relative abundance of microbial taxa that are known to be involved in nitrogen transformation processes such as nitrogen fixation, nitrification, and denitrification^30, 35, 36^. Other studies revealed that soil biochar amendment can significantly increase typical and atypical *nosZ* gene and transcript copies suggesting a higher abundance and activity of N_2_O reducers in response to soil biochar amendment^31, 32, 34, 37, 38^. Furthermore, it has been reported in a recent sequencing study, targeting typical and atypical *nosZ* genes, that biochar incorporation into soil can cause taxonomic shifts among microbes that carry the genetic potential for N_2_O reduction^33^. However, the impact of soil biochar amendment on the diversity and taxonomic composition of active denitrifiers and N_2_O reducing microbial taxa has not yet been systematically investigated on the transcript level.

In order to investigate biochar-induced changes in diversity and taxonomic composition of active denitrifiers and N_2_O reducers, we amplified and sequenced *nirK*, typical *nosZ*, and atypical *nosZ* transcripts in samples collected at different time points of sampling (0, 4, and 10 days) from a soil microcosm experiment that has been performed under denitrifying conditions^34^.

## Material and Methods

The RNA extracts analysed in this study were obtained from soil samples collected in a microcosm experiment previously described by Harter, *et al*.^34^. In the following we briefly describe the soil and biochar used for this soil microcosm experiment and its experimental design. For a detailed description of the experiment please refer to Harter, *et al*.^34^.

### Soil sampling and biochar production

The soil for the soil microcosm experiment was collected from the top 10 cm of an Anthrosol (World Reference Base for Soil Resources, 2014) located at an urban gardening site of the University of Tuebingen, Germany (48° 32′ N, 9° 4′ E). The soil is classified as sandy clay loam (USDA) with a particle size distribution of 49.8% sand, 25.8% silt and 24.4% clay. Biochar was produced by Swiss Biochar Sàrl (Belmont-sur-Lausanne, Switzerland) from green waste by slow pyrolysis (620 °C) according to the standards of the European Biochar Certificate (EBC) (http://www.european-biochar.org). Important physical and chemical properties of soil and biochar are summarized in Table 1. Detailed descriptions of the methods used to determine the physical and chemical properties of soil and biochar can be found in Harter, *et al*.^34^.

**Table 1:** Properties of soil and biochar.

property	unit	soil	biochar
pH_H2O_		7.59	9.18
C	%	3.42	73.2
CaCO_3_	%	5.03	na
C_org_	%	2.81	73.1
N	%	0.34	0.64
C_org_/N		8.26	114
H	%	na	1.03
O	%	na	5.70
H:C		na	0.17
O:C		na	0.058
Ash	%	na	19.4
EC	µS/cm	na	782
SA	m^2^/g	na	231
Ca	%	1.67	4.90
Fe	%	0.56	0.27
K	%	0.20	0.84
Mg	%	0.30	0.33
B	mg/kg	na	36
Cd	mg/kg	<0.2	<0.2
Cr	mg/kg	9.3	10
Cu	mg/kg	6.4	16
Hg	mg/kg	na	<0.07
Mn	mg/kg	109	310
Mo	mg/kg	<0.1	na
Na	mg/kg	13	830
Ni	mg/kg	6	8
P	mg/kg	208	1400
Pb	mg/kg	8	<2
S	mg/kg	228	400
Si	mg/kg	119	22000
Zn	mg/kg	32	45
PAHs	mg/kg	na	6.70

Table from Harter, *et al*.^34^. EC: electrical conductivity, SA: surface area, PAHs: polycyclic aromatic hydrocarbons (sum of the EPA’s 16 priority pollutants), na: not analysed.

### Soil microcosm experiment

Prior to microcosm setup the field moist (water content: 29% w/w) soil and the biochar were passed through a 2 mm sieve and thoroughly homogenized. Soil microcosms were set up in 250 ml glass beakers. Microcosms contained either the field moist equivalent of 100 g dry soil (control microcosms) or the field moist equivalent of 95 g dry soil and 5 g dry biochar resulting in a final biochar content of 5% (biochar microcosms). Control and biochar microcosms were set up in triplicates. After preparation, soil microcosms were homogenized and compacted (10 repetitions of dropping a 125 g hammer from a height of 20 cm onto the soil surface). In order to simulate conventional fertilization, all soil microcosms were fertilized with a NH_4_NO_3_ solution at a rate of 332 mg N kg^−1^ (equivalent to 100 kg N ha^−1^ estimated based on the soil surface area in the microcosms). The amount of water added with the NH_4_NO_3_ solution was calculated to adjust the water filled pore space (WFPS) to 90%. While the experiment was running the soil microcosms were covered with a perforated aluminium foil to allow gas exchange with the ambient atmosphere and to decrease evaporation. Soil microcosms were incubated at a constant temperature of 20 °C and the WFPS was held constant by periodically replenishing the evaporated water. Soil microcosms were incubated for 10 days with sampling taking place after 0, 1, 2, 4, 7 and 10 days of incubation. To allow destructive soil sampling of a set of 6 microcosms (3 control and 3 biochar microcosms) at each time point of sampling, we set up 36 soil microcosms in total. During soil sampling the entire soil of the soil microcosm was transferred into a sterile container, homogenized using a spatula and aliquoted for parallel RNA extraction. All samples for RNA extraction contained the equivalent of 2 g dry soil and were directly frozen at −80 °C. For the present sequencing study only RNA extracts obtained from soil samples collected at day 0, 4, and 10 were analysed.

### RNA extraction and reverse transcription

Total RNA was extracted using the RNA PowerSoil Total RNA Isolation Kit according to the manufacturer protocol (MO BIO Laboratories, Carlsbad, CA, USA). RNA concentration and quality of the resulting extracts were determined spectrophotometrically (NanoDrop 1000, Thermo Scientific, Waltham, MA, USA), fluorometrically (Qubit 2.0 Fluorometer, Life Technologies, Carlsbad, CA, USA), and by gel electrophoresis (Experion Automated Electrophoresis Station, Bio-Rad Laboratories, Hercules, CA, USA). Residual DNA in RNA extracts was digested using the Ambion TURBO DNA-free Kit (Life Technologies, Carlsbad, CA, USA) according to the instructions given by the manufacturer. Successful removal of DNA was confirmed by PCR using primers 27F (5′-AGAGTTTGATCMTGGCTCAG-3′)^39^ and PC5 (5′-TACCTTGTTACGACTT-3′)^40^ with the following conditions: hot start at 70 °C, 5 min at 95 °C, 35 cycles with 1 min at 95 °C, 1 min at 44 °C and 3 min at 72 °C followed by a final elongation step of 10 min at 72 °C. If the PCR revealed no PCR products, RNA extracts were used for cDNA synthesis. Reverse transcription of pure RNA extracts was performed with the SuperScript III Reverse Transcriptase using random primers according to the manufacturer’s protocol (Life Technologies, Carlsbad, CA, USA). cDNA samples were quality checked and quantified using agarose gel electrophoresis and Nanodrop (NanoDrop 1000, Thermo Scientific, Waltham, MA, USA).

### Illumina amplicon sequencing

In order to sequence *nirK*, typical *nosZ* and atypical *nosZ* mRNA transcripts we amplified the corresponding fragments from the cDNA samples from the control and biochar microcosms at day 0, 4 and 10 (n = 18) using PCR. PCRs were performed using target-specific primers fused with overhang adapter sequences at the 5′-end to allow the addition of index sequences and sequencing adapters in a second index PCR. Transcripts of *nirK* genes were amplified using primer F1aCu (5′-ATCATGGTSCTGCCGCG-3′) and R3Cu (5′-GCCTCGATCAGRTTGTGGTT-3′)^41^. For typical *nosZ* gene transcripts primer nosZ2F (5′-CGCRACGGCAASAAGGTSMSSGT-3′) and nosZ2R (5′- CAKRTGCAKSGCRTGGCAGAA-3′)^42^ were used. Transcripts of atypical *nosZ* genes were amplified with the primers nosZ-II-F (5′-CTNGGNCCNYTKCAYAC-3′) and nosZ-II-R (5′-GCNGARCARAANTCBGTRC-3′) developed by Jones, *et al*.^26^. PCRs were performed with the FastStart High Fidelity PCR system (Roche Diagnostics, Rotkreuz, Switzerland) using the thermal protocols and reaction mixtures described in Table S1 in the supplementary information. The produced amplicons were purified using AMPure XP beads (Beckman Coulter, Brea, CA, USA) at a ratio of 1:1 (v/v). Quality and quantity of the purified amplicons were determined using agarose gel electrophoresis and Nanodrop (NanoDrop 1000, Thermo Scientific, Waltham, MA, USA). Subsequent library preparation steps and sequencing were performed by IMGM Laboratories GmbH (Martinsried, Germany) according to the amplicon sequencing guidelines given by Illumina (San Diego, CA, USA). Sequencing was performed on an Illumina MiSeq sequencing system (Illumina, San Diego, CA, USA) using the 2 × 300 bp MiSeq Reagent Kit v3 (600 cycle) (Illumina, San Diego, CA, USA). The MiSeq Reporter Software v2.5.1.3 (Illumina, San Diego, CA, USA) was used for signal processing, de-multiplexing and trimming of adapter sequences.

### Sequence analysis

Quality control of raw *nirK*, typical *nosZ* and atypical *nosZ* read pairs was performed using Cutadapt v1.9^43^ and USEARCH v8.1.1812^44, 45^. At first target-specific primers were detected and trimmed using Cutadapt. Afterwards, paired-end *nirK* and typical *nosZ* reads were merged using the fastq_mergepairs algorithm implemented in USEARCH. Due to the large size of atypical *nosZ* amplicons (>600 bp) paired-end reads could not be merged. Instead, the forward read was trimmed to 200 bp to ensure the removal of bases with low phred scores and to obtain sequences of equal length. Trimming was performed with fastx_truncate using USEARCH. Merged (*nirK* and typical *nosZ*) and trimmed (atypical *nosZ*) sequences were quality filtered using the USEARCH fastq_filter function with a maximum error rate threshold of 1%. Chimeric sequences were de novo detected and removed using the uchime_denovo algorithm implemented in USEARCH^46^.

All quality filtered non-chimeric *nirK*, typical *nosZ* and atypical *nosZ* sequences were mapped against the NCBI Reference Sequence (RefSeq) protein database (release 66). Mapping was performed using DIAMOND (v0.7.9.58)^47^ in blastx mode with a minimum protein sequence identity cut-off of 70% and an e-value cut-off of 10^−10^. The top 50 database hits of each sequence were used for further analysis with MEGAN6 Ultimate Edition^48, 49^. For taxonomic placement the Lowest Common Ancestor (LCA) algorithm of MEGAN was applied^50^. The LCA analysis parameters ‘Top Percent’ and ‘Min Support’ were set to 0.5% (all hits within the top 0.5% of the best bit score are considered) and 10 (taxa need to obtain a minimum of 10 reads to be considered), respectively. Shannon-Weaver and Simpson-Reciprocal diversity indices were computed on the species level in MEGAN.

Absolute sequence counts and relative sequence abundances of all assigned taxa were exported from MEGAN for further analyses. Relative sequence abundances were calculated based on all sequences that matched database entries in the RefSeq protein database.

Typical and atypical *nosZ* transcript copy numbers of individual species at day 4 were calculated by multiplying absolute transcript copy numbers determined by qPCR in a previous study conducted by Harter, *et al*.^34^ with the corresponding relative sequence abundances of individual *nosZ*-expressing species obtained in this study. This was possible because sequencing and qPCRs were performed on the same RNA samples with the same sets of primers in the present and the previous study by Harter, *et al*.^34^. Individual species with an average relative sequence abundance below 1%, were not considered.

**Figure 1:**
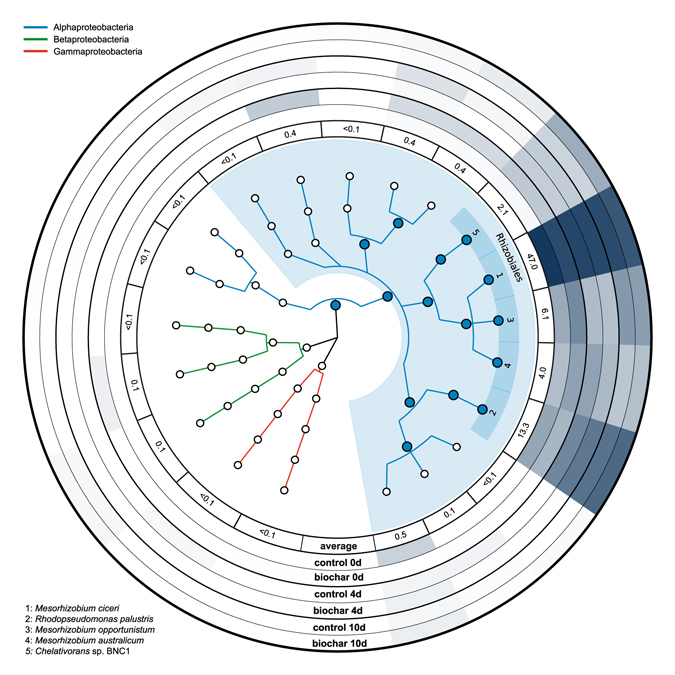
Circular taxonomic tree of *nirK*-expressing taxa. Microbial taxa are indicated by circles. Different classes are shown with specific branch colours. Large, filled, circles depict microbial taxa with average relative sequence abundances above 1%. Light colour shaded areas indicate orders with a relative sequence abundance above 1%. Dark colour shaded boxes with numbers (1–5) represent species with a relative sequence abundance above 1%. External rings show the relative sequence abundance of the corresponding species. Ring 1 shows the average relative sequence abundance in percent (all microcosms at all time points of sampling, n = 18). Rings 2–7 show the relative sequence abundances in control and biochar microcosms at day 0, 4, and 10 as circular heatmaps (n = 3).

**Figure 2:**
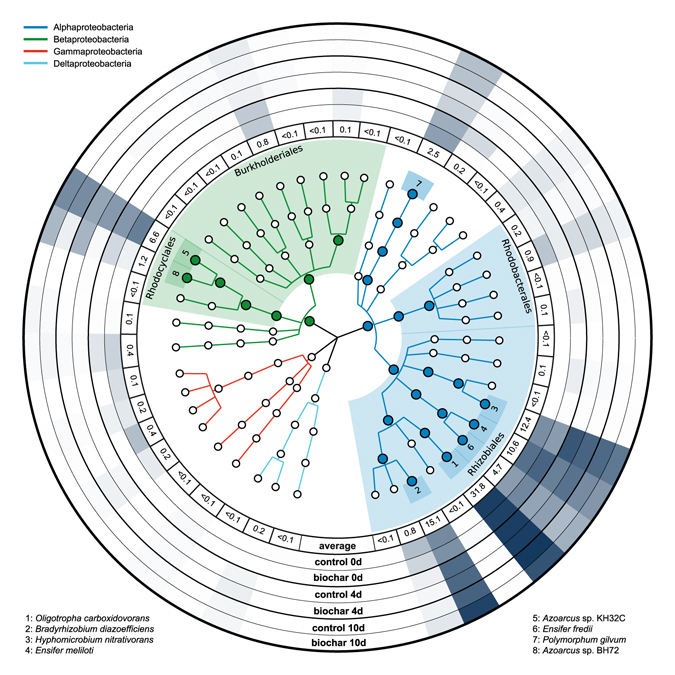
Circular taxonomic tree of typical *nosZ*-expressing taxa. Microbial taxa are indicated by circles. Different classes are shown with specific branch colours. Large, filled, circles depict taxa with average relative sequence abundances above 1%. Light colour shaded areas indicate orders with a relative sequence abundance above 1%. Dark colour shaded boxes with numbers (1–8) represent species with a relative sequence abundance above 1%. External rings show the relative sequence abundance of the corresponding species. Ring 1 shows the average relative sequence abundance in percent (all microcosms at all time points of sampling, n = 18). Rings 2–7 show the relative sequence abundances in control and biochar microcosms at day 0, 4, and 10 as circular heatmaps (n = 3).

**Figure 3:**
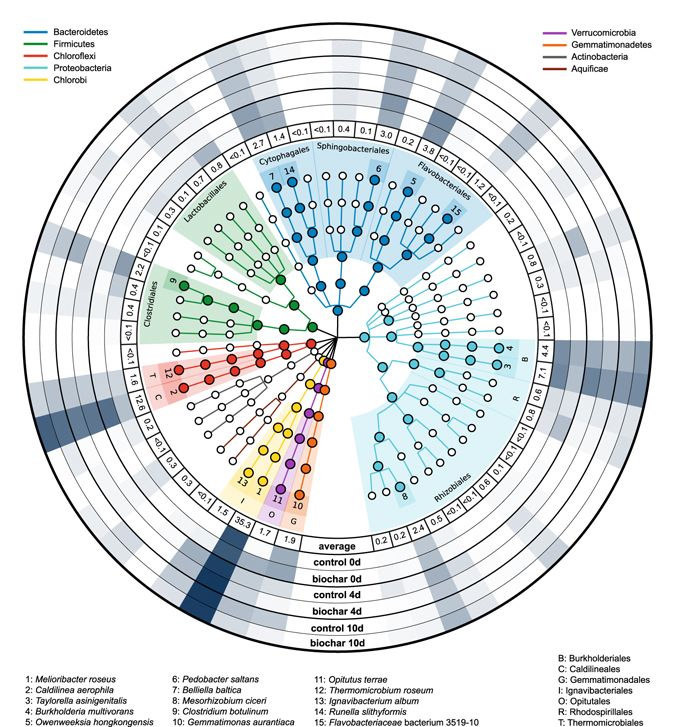
Circular taxonomic tree of the atypical *nosZ*-expressing taxa. Microbial taxa are indicated by circles. Different phyla are shown with specific branch colours. Large, filled circles depict taxa with average relative sequence abundances above 1%. Light colour shaded areas indicate orders with a relative sequence abundance above 1%. Dark colour shaded boxes with numbers (1–15) represent species with a relative sequence abundance above 1%. External rings show the relative sequence abundance of the corresponding species. Ring 1 shows the average relative sequence abundance in percent (all microcosms at all time points of sampling, n = 18). Rings 2–7 show the relative sequence abundances in control and biochar microcosms at day 0, 4, and 10 as circular heatmaps (n = 3).

Figures 1, 2 and 3 were generated with GraPhlAn^51^ using the LCA-based taxonomy and the corresponding relative sequence abundance information from MEGAN. According to the basic principle of the LCA algorithm, sequences that are conserved among different species (e.g. as consequence of horizontal gene transfer) will only be assigned to taxa of higher rank^50^. Nonetheless, it is very difficult to directly prove that a given *nirK* or *nosZ* gene (typical and atypical) appears in a specific microbial taxon. Thus, whenever we mention specific taxa in the results and discussion we refer to a taxonomic group of microorganisms that contains a *nirK* or *nosZ* gene closely related to the *nirK* or *nosZ* gene of the respective taxa.

### Statistical analysis

**Figure 4:**
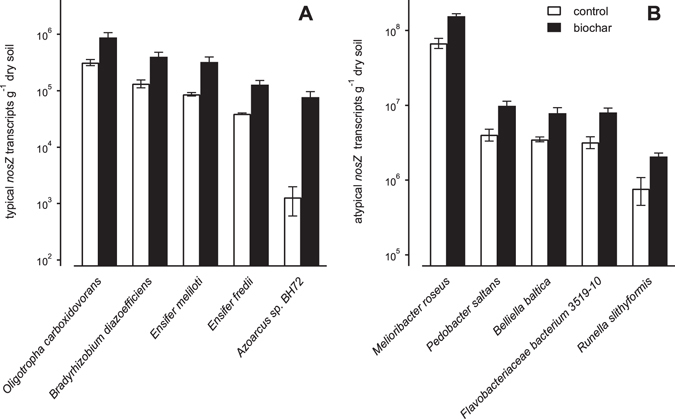
Calculated absolute typical (**A**) and atypical (**B**) *nosZ* transcript copy numbers originating from specific species in control (white bars) and biochar (black bars) microcosms at day 4. The figure shows only species where biochar addition had a statistically significant effect on transcript production (*p* < 0.05). Bars and error indicators represent means and standard errors (n = 3), respectively.

The effects of biochar addition on diversity indices (Tables 2 and 3) and the relative sequence abundance of each assigned microbial species (Figs 1,2,3), were statistically evaluated at each time point of sampling (0, 4, and 10 days) using t-tests. In addition, we used t-tests to determine significant biochar effects on the absolute *nosZ* transcript copy numbers of individual species at day 4 (Fig. 4). Differences in diversity indices among the average (all control and biochar microcosms at all time points of sampling, n = 18) *nirK*, typical *nosZ* and atypical *nosZ*-expressing microbial communities (Tables 2 and 3) were determined using one-way ANOVA with Tukey’s HSD post-hoc test. T-tests and one-way ANOVAs were carried out in SAS (SAS 9.2, SAS Institute, Cary, NC, USA) using PROC TTEST and PROC GLM, respectively. Dissimilarities among *nirK*, typical *nosZ* and atypical *nosZ*-expressing microbial communities in control and biochar microcosms and over time were statistically evaluated using permutational multivariate analysis of variance (PERMANOVA) with biochar addition and time as main factors and 10^5^ permutations (Table 4). PERMANOVAs were performed based on Bray-Curtis dissimilarity matrices using the adonis function implemented in the R package vegan^52^.

**Table 2:** Shannon-Weaver diversity index for *nirK*, typical *nosZ*, and atypical *nosZ*, in control and biochar microcosms after 0, 4, and 10 days.

	*nirK*			typical *nosZ*			atypical *nosZ*		
	**control**	biochar	sig.	control	biochar	sig.	control	biochar	sig.
**day 0**	0.99 ± 0.47	1.77 ± 0.32	ns	3.04 ± 0.11	2.90 ± 0.03	ns	3.49 ± 0.37	3.46 ± 0.25	ns
**day 4**	1.49 ± 0.29	1.61 ± 0.06	ns	2.60 ± 0.05	2.73 ± 0.03	*	3.23 ± 0.17	2.96 ± 0.08	ns
**day 10**	1.59 ± 0.03	1.92 ± 0.07	**	2.50 ± 0.11	2.66 ± 0.02	ns	3.08 ± 0.37	2.97 ± 0.16	ns
**average**	1.56 ± 0.37^a^			2.74 ± 0.20^b^			3.20 ± 0.31^c^		

“average” shows average values of all microcosms at all time points of sampling. Values represent means ± standard deviation (individual samples: n = 3, average: n = 18). Significant differences between individual control and biochar samples are shown in the column “sig.” (ns = not significant, *p < 0.05, **p < 0.01). Significant differences (*p* < 0.05) between the average values of *nirK*, typical *nosZ* and atypical *nosZ* are indicated by superscripted letters next to the corresponding values.

**Table 3:** Simpson-Reciprocal diversity index for *nirK*, typical *nosZ*, and atypical *nosZ*, in control and biochar microcosms after 0, 4, and 10 days.

	*nirK*			typical *nosZ*			atypical *nosZ*		
	control	biochar	sig.	control	biochar	sig.	control	biochar	sig.
**day 0**	1.53 ± 0.34	2.51 ± 0.91	ns	5.52 ± 0.70	4.82 ± 0.29	ns	6.97 ± 2.76	6.92 ± 1.00	ns
**day 4**	2.07 ± 0.35	2.18 ± 0.13	ns	4.47 ± 0.14	4.84 ± 0.07	*	5.03 ± 0.29	3.85 ± 0.37	*
**day 10**	2.37 ± 0.14	2.96 ± 0.08	**	4.03 ± 0.34	4.62 ± 0.14	*	4.24 ± 0.63	4.28 ± 0.44	ns
**average**	2.27 ± 0.58^a^			4.72 ± 0.55^b^			5.21 ± 1.68^b^		

“average” shows average values of all microcosms at all time points of sampling. Values represent means ± standard deviation (individual samples: n = 3, average: n = 18). Significant differences between individual control and biochar samples are shown in the column “sig.” (ns = not significant, *p < 0.05, **p < 0.01). Significant differences (*p* < 0.05) between the average values of *nirK*, typical *nosZ* and atypical *nosZ* are indicated by superscripted letters next to the corresponding values.

**Table 4:** Results from Bray-Curtis dissimilarity based two-way PERMANOVAs.

	*nirK*			typical *nosZ*			atypical *nosZ*		
	F	R^2^	*p*	F	R^2^	*p*	F	R^2^	*p*
**biochar**	3.26	0.10	0.051	3.08	0.11	**0**.**017**	1.31	0.06	0.222
**time**	14.5	0.46	**<0**.**001**	8.74	0.32	**<0**.**001**	5.18	0.24	**<0**.**001**
biochar * time	0.77	0.02	0.461	1.71	0.06	0.156	1.37	0.06	0.205

The table shows pseudo F-statistics (F), explained variances (R^2^), and the level of significance (*p*) for the two main effects “biochar” and “time” and their interaction “biochar*time”. Significant effects indicated by *p*-values below 0.05 are shown in bold font.

### Data availability

Illumina sequencing reads have been deposited in the ENA Sequence Read Archive (SRA) under accession number PRJEB14348.

## Results

### Sequencing statistics

Transcript sequencing resulted in 3,404,618 raw pairs of reads in total with *nirK*, typical *nosZ*, and atypical *nosZ* accounting for 733,518 (40,751 ± 13,287 per sample), 2,064,131 (114,674 ± 35,377 per sample), and 606,969 (33,721 ± 9,523 per sample) raw read pairs, respectively.

Quality processing resulted in 375,665 merged high quality *nirK* sequences of which 374,783 (20,821 ± 8,672 per sample) matched entries in the RefSeq database (protein sequence identity cut-off: 70%, e-value cut-off: 10^−10^). Merged high quality *nirK* sequences had an average sequence length of 436 bp.

For typical *nosZ*, 1,456,124 merged high quality sequences remained after quality processing and 1,420,025 (78,890 ± 24,006 per sample) matched database entries (protein sequence identity cut-off: 70%, e-value cut-off: 10^−10^). The average sequence length was 223 bp.

Quality processing of atypical *nosZ* sequences led to 288,167 high quality sequences with a sequence length of 200 bp. 166,786 sequences (9266 ± 2243 per sample) matched database entries (protein sequence identity cut-off: 70%, e-value cut-off: 10^−10^).

### Diversity of the microbial communities expressing *nirK*, typical *nosZ*, and atypical *nosZ*

In order to investigate the impact of soil biochar amendment on the diversity of expressed *nirK* and *nosZ* genes, we computed Shannon-Weaver and Simpson-Reciprocal diversity indices for all samples.

Shannon-Weaver indices ranged from 0.99 to 3.49 and were generally highest for atypical *nosZ* and lowest for *nirK* (Table 2). On average (all control and biochar microcosms at all sampling time points, n = 18), the *nirK*-expressing community had a value of 1.56. In comparison with *nirK*, typical and atypical *nosZ*-expressing communities showed significantly higher values of 2.74 (*p* < 0.001) and 3.20 (*p* < 0.001), respectively. Atypical *nosZ* had a significantly higher average Shannon-Weaver index than typical *nosZ* (*p* < 0.001). Biochar addition altered the Shannon-Weaver index at all time points of sampling. For *nirK* and typical *nosZ* a biochar-induced increase in diversity was observed (Table 2). Atypical *nosZ* diversity slightly decreased in the presence of biochar. While this slight decrease was not statistically significant at any time point of sampling, the values for *nirK* at day 10 (*p* = 0.002) and typical *nosZ* at day 4 (*p* = 0.014) were significantly higher in biochar compared to control microcosms.

The Simpson-Reciprocal diversity index showed a very similar pattern and ranged from 1.53 to 6.97 (Table 3). With an average value of 5.21, the highest diversity was determined for the atypical *nosZ*-expressing microbial community. The average value for typical *nosZ* was slightly, but not significantly, lower (4.72). However, with a value of 2.27 the *nirK*-expressing community had a significantly lower average diversity compared to the microbial communities producing typical (*p* < 0.001) and atypical (*p* < 0.001) *nosZ* transcript. Biochar addition significantly increased the Simpson-Reciprocal diversity index of typical *nosZ* transcripts at day 4 (*p* = 0.016) and 10 (*p* = 0.049) (Table 3). In addition, significantly higher values in biochar compared to control microcosms were also determined for *nirK* at day 10 (*p* = 0.003). For atypical *nosZ* soil biochar amendment resulted in significantly lower values at day 4 (*p* = 0.012).

In order to determine if biochar addition or time caused significant dissimilarities among *nirK*, typical *nosZ* and atypical *nosZ*-expressing microbial communities, we performed two-way PERMANOVAs. As shown in Table 4, PERMANOVAs revealed a significant time effect for *nirK* (*p* < 0.001), typical *nosZ* (*p* < 0.001) and atypical *nosZ*-expressing microbial communities (*p* < 0.001) and a significant biochar effect for the typical *nosZ*-expressing microbial community (*p* = 0.017).

### Classification of *nirK* transcripts

Regardless of the time point of sampling and the type of microcosm (biochar or control) all microbial taxa that produced copper nitrite reductase gene (*nirK*) transcripts were affiliated with the phylum Proteobacteria (Fig. 1). 99.8% of all *nirK* sequences belonged to the order Rhizobiales. 74.4% could be classified down to the species level with *Mesorhizobium ciceri* (47.0%), *Rhodopseudomonas palustris* (13.3%), *Mesorhizobium opportunistum* (6.12%), *Mesorhizobium australicum* (4.02%), and *Chelativornas* sp. BNC1 (2.05%) showing a relative sequence abundance above 1% (Fig. 1). A complete list with the relative sequence abundances of all *nirK*-expressing microbial species identified in this study can be found in Table S2 in the supplementary information.

The taxonomic composition and distribution of *nirK* transcripts slightly differed in control and biochar microcosms at all time points of sampling (0, 4, and 10 days) (Fig. 1). However, biochar related changes in relative sequence abundance of specific species did not follow a clear trend. Accordingly, significantly higher and lower relative sequence abundances in biochar compared to control microcosms were only detected for *Mesorhizobium australicum* at day 0 (control: 3.0%, biochar: 6.3%, *p* = 0.048) and *Mesorhizobium ciceri* at day 10 (control: 44.0%, biochar: 33.6%, *p* = 0.011), respectively (Fig. 1).

### Classification of typical *nosZ* transcripts

Similar to *nirK*, all *nosZ* transcripts were affiliated with the phylum Proteobacteria (Fig. 2). With a relative sequence abundance of 77.1%, most sequences belonged to the order Rhizobiales. Rhodocyclales, Burkholderiales, and Rhodobacterales had relative sequence abundances of 7.8%, 6.9%, and 1.2%, respectively. In total, 90.2% of all typical *nosZ* sequences could be classified down to the species level. The most abundant species were: *Oligotropha carboxidovorans* (31.8%), *Bradyrhizobium diazoefficiens* (15.1%), *Hyphomicrobium nitrativorans* (12.4%), *Ensifer meliloti* (10.6%), Azoarcus sp. KH32C (6.57%), *Ensifer fredii* (4.68%), *Polymorphum gilvum* (2.45%), and *Azoarcus* sp. BH72 (1.24%). All of these species had a relative sequence abundance above 1% (Fig. 2). A complete list with the relative sequence abundances of all typical *nosZ*-expressing microbial species identified in this study can be found in Table S3 in the supplementary information.

In agreement with the PERMANOVA results, the taxonomic composition and distribution of typical *nosZ* transcripts was significantly affected by biochar addition (Fig. 2). Although the contribution of certain species to total typical *nosZ* transcripts was different in control and biochar microcosms throughout all time points of sampling (0, 4 and 10 days), statistically significant differences in relative sequence abundance were only determined after 4 and 10 days of incubation. At day 4, soil biochar amendment significantly increased the relative sequence abundance of *Ensifer meliloti* (control: 9.3%, biochar: 12.2%, *p* = 0.019) and *Azoarcus* sp. BH72 (control: 0.1%, biochar: 2.9%, *p* = 0.010). The relative sequence abundances of *Hyphomicrobium nitrativorans* (control: 20.6%, biochar: 16.1%, *p* = 0.002) and *Azoarcus* sp. KH32C (control: 5.4%, biochar: 4.1%, *p* = 0.048) were significantly lower in the presence of biochar (Fig. 2). After 10 days of incubation, significantly higher relative sequence abundances in biochar compared to control microcosms were determined for *Bradyrhizobium diazoefficiens* (control: 16.9%, biochar: 30.3%, *p* = 0.025), *Ensifer fredii* (control: 2.6%, biochar: 3.3%, *p* = 0.021), and *Azoarcus* sp. BH72 (control: 0.4%, biochar: 2.7%, *p* = 0.021). Significantly lower values were found for *Oligotropha carboxidovorans* (control: 36.8%, biochar: 22.2%, *p* = 0.006) (Fig. 2).

### Classification of atypical *nosZ* transcripts

Atypical *nosZ*-expressing microorganisms belonged to a broad diversity of different phyla (Fig. 3). About 36.9% of all sequences were affiliated with the phylum Chlorobi. Other important phyla were: Proteobacteria (19.5%), Bacteroidetes (15.1%), Chloroflexi (14.2%), Firmicutes (5.6%), Gemmatimonadetes (1.9%), and Verrucomicrobia (1.7%). Sequences of atypical *nosZ* transcripts were classified into 25 orders with Ignavibacteriales (36.9%), Caldilineales (12.6%), and Burkholderiales (11.6%) representing the most abundant taxa.

Also, Flavobacteriales (5.3%), Rhizobiales (4.7%), Cytophagales (4.6%), Sphingobacteriales (3.7%), Clostridiales (3.3%), Lactobacillales (2.0%), Gemmatimonadales (1.9%) Opitutales (1.7%), Thermomicrobiales (1.6%), and Rhodospirillales (1.4%) were detected at relative sequence abundances above 1% (Fig. 3). In total, 91.6% of all atypical *nosZ* sequences could be classified to taxa at the species level. 15 species had relative sequence abundances above 1%: *Melioribacter roseus*, *Caldilinea aerophila*, *Taylorella asinigenitalis*, *Burkholderia multivorans*, *Owenweeksia hongkongensis*, *Pedobacter saltans*, *Belliella baltica*, *Mesorhizobium ciceri*, *Clostridium botulinum*, *Gemmatimonas aurantiaca*, *Opitutus terrae*, *Thermomicrobium roseum*, *Ignavibacterium album*, *Runella slithyformis*, and *Flavobacteriaceae* bacterium 3519-10 (Fig. 3). A complete list with the relative sequence abundances of all atypical *nosZ*-expressing microbial species identified in this study can be found in Table S4 in the supplementary information.

The impact of soil biochar amendment on the taxonomic composition and distribution of atypical *nosZ* transcripts was minor. Although differences in relative sequence abundance between control and biochar microcosms were observed for several species at all time points of sampling (0, 4 and 10 days), most of them were not statistically significant. Significantly higher relative sequence abundances in biochar compared to control microcosms were determined for *Ignavibacterium album* at day 0 (control: 0%, biochar: 0.3%, *p* = 0.035) and 10 (control: 0.7%, biochar: 1.5%, *p* = 0.039) and for *Melioribacter roseus* at day 4 (control: 37.2%, biochar: 43.7%, *p* = 0.015). A biochar-induced statistically significant decrease was not observed.

### Typical and atypical *nosZ* transcript copies of individual species at day 4

At day 4, soil biochar amendment significantly altered the calculated abundance of typical *nosZ* transcripts in 5 relevant (relative sequence abundance >1%) species. The calculated number of typical *nosZ* transcript copies of the following species was significantly increased in the presence of biochar (Fig. 4A): *Oligotropha carboxidovorans* (*p* = 0.042), *Bradyrhizobium diazoefficiens* (*p* = 0.028), *Ensifer meliloti* (*p* = 0.028), *Ensifer fredii* (*p* = 0.021), and *Azoarcus* sp. BH72 (*p* = 0.016). Transcript copy numbers originating from other species were not significantly affected by biochar addition.

Similar to typical *nosZ*, significant biochar-induced changes in the production of atypical *nosZ* transcripts at day 4 were determined for only 5 (relative sequence abundance >1%) species (Fig. 4B). Calculated atypical *nosZ* transcript copy numbers of *Melioribacter roseus* (*p* = 0.005), *Pedobacter saltans* (*p* = 0.024), *Belliella baltica* (*p* = 0.041), *Runella slithyformis* (*p* = 0.029), and *Flavobacteriaceae* bacterium 3519-10 (*p* = 0.019) were significantly higher in biochar compared to control microcosms. Absolute transcript production of other atypical *nosZ*-expressing species was not significantly altered by soil biochar amendment.

## Discussion

Highest N_2_O emission rates usually occur after heavy rain fall when high amounts of organic carbon and mineral nitrogen are mobilized and oxygen availability decreases due to high water filled pore spaces (WFPS)^17, 18^. These conditions promote the growth and activity of microbial strains capable of performing anaerobic nitrogen transformation processes such as denitrification^19, 20^. In order to simulate such conditions, we set up a soil microcosm experiment at a constant WFPS of 90% to which we added a NH_4_NO_3_ solution at concentrations that reflect common agricultural application rates. Although the amount of N_2_O emitted under these conditions can account for large fractions of total annual emissions, a high WFPS is usually occurring only a few days after rain events in most soils^17, 18^. Accordingly, our experiment was designed to mimic the frequently observed high N_2_O emissions occurring in response to soil water saturation over a short time period (10 days of incubation, 6 time points of sampling).

Harter, *et al*.^34^ described and discussed the impact of biochar addition on nitrogen transformation rates, absolute gene and transcript copy numbers of denitrification marker genes, and the formation and release of N_2_O and N_2_. According to Harter, *et al*.^34^, biochar addition significantly decreased N_2_O emissions and increased transcript copy numbers of specific functional denitrification genes. While transcripts of the denitrification genes *napA*, *narG* and *nirS* did not show significant biochar effects or were subject to a significant interaction effect (biochar*time), *nirK*, typical *nosZ* and atypical *nosZ* transcript copy numbers were significantly increased by biochar addition. Thus, the current Illumina amplicon sequencing study described here focuses on biochar-induced changes in diversity and relative abundance among the microbial communities actively expressing *nirK*, typical *nosZ* and atypical *nosZ* transcripts.

According to both diversity indices (Shannon-Weaver and Simpson-Reciprocal) the *nirK*-expressing microbial community had a significantly lower diversity than the microbial communities producing typical *nosZ* and atypical *nosZ* transcripts. A lower diversity of the *nirK*-harbouring community compared to the microbial community carrying typical *nosZ* has also been reported by Palmer and Horn^53^. The microbial community that actively expressed atypical *nosZ* genes was significantly more diverse (Shannon-Weaver diversity index) than the community expressing typical *nosZ* genes. These findings confirm the results of Jones, *et al*.^29^ who determined an up to 3-fold higher Faith’s phylogenetic diversity (PD) for atypical *nosZ* compared to typical *nosZ* genes. In accordance with our results, Jones and colleagues also demonstrated a significant relationship between the relative abundance and the phylogenetic diversity of the *nosZ* community and the ability of the soil microbial community to reduce N_2_O. Using network and co-occurrence analyses, Jones, *et al*.^29^ found that functional groups that were identified as significant indicators of reduced soil N_2_O emissions were dominated by atypical *nosZ* communities. Notably, Jones and colleagues found specific atypical *nosZ* groups in high abundance in soils for which the lowest N_2_O emissions were observed. These atypical *nosZ* lineages lacked either *nir* genes but seemed to be critical for the N_2_O sink capacity of the respective soil. Interestingly, the observation made by Jones, *et al*.^29^ with respect to *nirK* and *nosZ* gene abundance and diversity are very similar to what we report here for a biochar-amended soil and *nirK* and *nosZ* transcript abundance and diversity under conditions of reduced N_2_O emissions.

The taxonomic compositions of the microbial communities expressing *nirK*, typical *nosZ*, and atypical *nosZ* described here, were in good agreement with other sequencing studies performed on soil. In accordance with the findings of Bremer, *et al*.^54^ and Henry, *et al*.^55^, the *nirK*-expressing community was largely dominated by the order Rhizobiales and the genus *Mesorhizobium*. The typical *nosZ*-expressing soil community was dominated by Alpha- and Betaproteobacteria and species affiliated with the genera *Oligotropha*, *Bradyrhizobium*, *Hyphomicrobium*, *Ensifer*, and *Azoarcus*. The importance of these genera within the typical *nosZ* gene-carrying community, as well as the dominance of microbial taxa that belong to the classes Alpha- and Betaproteobacteria has been reported in several previous soil studies^26, 28, 29, 33, 53, 56^. While all *nirK* and typical *nosZ* transcripts were produced by microbial taxa affiliated with the phylum Proteobacteria, the atypical *nosZ*-expressing community comprised a board variety of different microbial species affiliated with several phyla including Bacteroidetes, Chlorobi, Chloroflexi, and Firmicutes. These results support the findings of Jones, *et al*.^26^ and Sanford, *et al*.^27^ who showed that N_2_O reduction is a widespread functional trait among soil microbes. Furthermore, the determined species reflected the taxonomic composition of atypical *nosZ*-carrying microbial species identified in similar sequencing studies and a recent soil metagenome analysis^28, 29, 33^.

Soil biochar amendment significantly increased the diversity indices computed for *nirK* (day 10) and typical *nosZ* (day 4 and 10). Higher diversity indices in biochar-amended compared to biochar-free soils have been reported frequently in studies targeting 16S rRNA genes^31, 35, 57^. The reason for the biochar-induced increase in microbial diversity is mostly unknown. However, as mentioned in other studies, it seems likely that organic compounds attached to the biochar particles^58, 59^, the high pH of biochar-amended soils^35^, or other biochar properties (surface area, pore space) promoted the formation of specific niches, which supported the growth and activity of a diverse range of taxa^33, 35, 57, 60^.

PERMANOVA analyses indicated that the composition of *nirK*, typical *nosZ* and atypical *nosZ*-expressing microbial communities were significantly altered over the whole duration of the experiment (p < 0.001) and that the composition of typical *nosZ* transcripts was significantly affected by biochar addition (p < 0.017). Among the typical *nosZ*-expressing community biochar addition resulted in a significant increase in relative abundance of *Ensifer meliloti* (day 4), *Azoarcus* sp. BH72 (day 4 and 10), *Bradyrhizobium diazoefficiens* (day 10), and *Ensifer fredii* (day 10). Interestingly all of these microbial species are primarily known for their ability to perform nitrogen fixation and usually form a symbiotic relationship with legumes or grasses^61–64^. Except for *Azoarcus* sp. BH72, which does not contain *nirK* or *nirS* genes, all species carry the full set of denitrification genes (*napA*/*narG*, *nirK*/*nirS*, *norB*, *nosZ*)^65^. Furthermore, it is known that many Rhizobia species are able to denitrify in their free-living states as well as in association with legume root nodules^66^. Bacterial strains affiliated with the genera *Ensifer*, *Azoarcus*, and *Bradyrhizobium* are ubiquitous in soils and have been identified in numerous 16S rRNA and typical *nosZ* gene-based sequencing studies in natural and biochar-amended soils^33, 35, 53, 67^.

In order to further our understanding of the effects biochar has on microbial N_2_O reduction, we calculated the absolute numbers of typical and atypical transcript copies produced by individual species based on the qPCR data from Harter, *et al*.^34^ and the relative transcript abundances determined in the present sequencing study. Species-specific transcript expression was specifically determined at day 4, because biochar most significantly affected total transcript copy numbers and N_2_O emission rates at this day^34^.

Biochar addition to soil significantly increased the calculated production of *nosZ* transcripts in 5 typical and 5 atypical *nosZ*-carrying species. Typical *nosZ* transcript production was enhanced in *Oligotropha carboxidovorans*, *Bradyrhizobium diazoefficiens*, *Ensifer meliloti*, *Ensifer fredii*, and *Azoarcus* sp. BH72. An increased transcription of atypical *nosZ* genes in biochar microcosms was determined for *Melioribacter roseus*, *Pedobacter saltans*, *Belliella baltica*, *Flavobacteriaceae* bacterium 3519-10, and *Runella slithyformis*. The calculated production of typical and atypical *nosZ* transcripts in all other relevant microbial species we identified based on the sequencing of typical and atypical *nosZ* transcripts was not significantly affected by biochar addition. Recent studies revealed that well-studied strains of some of these species (*Ensifer meliloti* 1021 and *Bradyrhizobium diazoefficiens* USDA 110) are able to grow with externally supplied N_2_O as sole electron acceptor^68, 69^. Thus, these strains take up N_2_O from the environment and further reduce it to N_2_. This is in contrast to other denitrifying strains such as *Pseudomonas aeruginosa* PAO1 which cannot grow on exogenous N_2_O as sole electron acceptor^70^. In addition to these species that are presumably able to physiologically specialize on N_2_O reduction many other species that had a significantly higher *nosZ* transcript production in biochar microcosms such as *Azoarcus* sp. BH72, *Flavobacteriaceae* bacterium 3519-10, *Belliella baltica*, *Melioribacter roseus*, *Pedobacter saltans*, and *Runella slithyformis* lack at least one of the functional genes encoding nitrate, nitrite, and nitric oxide reductases^65^. Thus these species are forced to perform a truncated version of the denitrification pathway by taking up denitrification intermediates (e.g. N_2_O) from the environment that have been produced by other denitrifiers^71^. Taken together these findings suggest that soil biochar amendment significantly increased *nosZ* transcript expression in microbial species capable of reducing exogenous N_2_O from the environment.

Our results are in good agreement with the findings from a recent DNA-based sequencing study in which biochar addition significantly increased relative sequence abundances of microbial species capable to use exogenous N_2_O as electron acceptor directly from the environment^33^. The cause for the increase in *nosZ* expression in these specific species might be due to the higher quantities of entrapped N_2_O in biochar microcosms^34^ that can serve as electron acceptor and thus lead to an increase in their transcriptional activity. Based on the high availability of N_2_O, species specialized on N_2_O reduction that are able to reduce exogenous N_2_O from the environment might gain a competitive advantage over species performing the full denitrification pathway considering the thermodynamic advantages associated with directly reducing N_2_O to N_2_^72^. However, although N_2_O entrapment in biochar-amended soils might be an important stimulant for increased *nosZ* gene expression in exogenous N_2_O reducers, we cannot exclude that other factors that are directly related to the physicochemical properties of biochar might have also contributed to the observed increase in *nosZ* transcription among the identified taxa. As reported in other studies several properties of biochar have the potential to significantly alter microbial communities by directly affecting microbial growth and activity of microorganisms (e.g. pH, biochar’s redox activity, nutrient sorption)^60, 73–76^. In our study biochar’s high pH might have stimulated alkaliphilic *Ensifer meliloti* and *Ensifer fredii* strains^61, 77^. Furthermore, it is possible that the high aromaticity of biochar or organic compounds that were attached to the biochar particles promoted *Bradyrhizobium diazoefficiens* strains that are able to degrade aromatic substances^78^.

Independent of the exact mechanism causing higher transcript production in these species, a higher N_2_O reduction activity of microbes that are potentially capable to reduce exogenous N_2_O from the environment will result in a decrease of the N_2_O/(N_2_O + N_2_) ratio. Thus, together with the findings from our previous study^34^, the decreased N_2_O emissions from biochar-amended soils are most likely caused by N_2_O entrapment resulting in retention and increased availability of N_2_O for microbial reduction in the water-filled pore space. The lower N_2_O/(N_2_O + N_2_) ratio is then caused by a higher microbial N_2_O reduction activity of *nosZ*-containing microorganisms able to directly reduce exogenous N_2_O from the environment.

In conclusion, this soil microcosm study showed that soil biochar amendment can lead to dynamic changes in the diversity of active denitrifier populations over time. Sequencing of *nirK*, typical *nosZ* and atypical *nosZ* transcripts revealed a significant biochar-induced increase in *nirK* and typical *nosZ* diversity and a higher relative sequence abundance of typical *nosZ*-expressing Rhizobia species. Furthermore, biochar addition led to a significantly higher calculated typical and atypical *nosZ* transcript expression among microbial species that are capable of directly reducing exogenous N_2_O from the environment. Our results suggest that the increased activity of these specific microbial species might be responsible for the observed dynamic changes in N_2_O/(N_2_O + N_2_) ratios in biochar vs control microcosms. We relate the observed increase in soil N_2_O sink capacity to the higher activity of specific *nosZ* gene taxa in response to N_2_O entrapment in the water-filled pore spaces of the biochar-amended soil. These findings further improve our understanding of the mechanisms responsible for biochar-induced N_2_O emission mitigation. However, it has to be taken into account that species-specific transcript expression was calculated based on qPCR and sequencing data collected from a short-term experiment performed in plant-free, fertilized soil microcosms with a high water content. Hence, generalizations and extrapolation to field scales and extended time periods should be done cautiously until the findings from the present study have been evaluated using different biochars and soils at spatial and temporal scales that more closely translate to field conditions.

## Electronic supplementary material

Supplementary Information

## References

[1] Thomson AJ, Giannopoulos G, Pretty J, Baggs EM, Richardson DJ (2012). Biological sources and sinks of nitrous oxide and strategies to mitigate emissions. Philos Trans R Soc, B.

[2] Hartmann, D. L. *et al*. In *Climate Change 2013*: *The Physical Science Basis*. *Contribution of Working Group I to the Fifth Assessment Report of the Intergovernmental Panel on Climate Change* (eds T. F. Stocker *et al*.) Ch. 2, 159–254 (Cambridge University Press, 2013).

[3] Ciais, P. *et al*. In *Climate Change 2013*: *The Physical Science Basis*. *Contribution of Working Group I to the Fifth Assessment Report of the Intergovernmental Panel on Climate Change* (eds T. F. Stocker *et al*.) Ch. 6, 465–570 (Cambridge University Press, 2013).

[4] Lehmann, J. & Joseph, S. Biochar for environmental management: science and technology. (Earthscan, 2009).

[5] Smith, P. *et al*. In *Climate Change 2014*: *Mitigation of Climate Change*. *Contribution of Working Group III to the Fifth Assessment Report of the Intergovernmental Panel on Climate Change* (eds O. Edenhofer *et al*.) Ch. 11, 811–922 (Cambridge University Press, 2014).

[6] Cayuela ML (2014). Biochar’s role in mitigating soil nitrous oxide emissions: A review and meta-analysis. Agr Ecosyst Environ.

[7] Hagemann, N. *et al*. Does soil aging affect the N_2_O mitigation potential of biochar? A combined microcosm and field study. *GCB Bioenergy*, doi:10.1111/gcbb.12390 (2016).

[8] Atkinson CJ, Fitzgerald JD, Hipps NA (2010). Potential mechanisms for achieving agricultural benefits from biochar application to temperate soils: a review. Plant Soil.

[9] Sohi SP (2012). Agriculture. Carbon storage with benefits. Science.

[10] Joseph SD (2010). An investigation into the reactions of biochar in soil. Aust J Soil Res.

[11] Singh B, Singh BP, Cowie AL (2010). Characterisation and evaluation of biochars for their application as a soil amendment. Aust J Soil Res.

[12] Kuzyakov Y, Bogomolova I, Glaser B (2014). Biochar stability in soil: Decomposition during eight years and transformation as assessed by compound-specific ^14^C analysis. Soil Biol Biochem.

[13] Jien SH, Wang CS (2013). Effects of biochar on soil properties and erosion potential in a highly weathered soil. Catena.

[14] Yao Y, Gao B, Zhang M, Inyang M, Zimmerman AR (2012). Effect of biochar amendment on sorption and leaching of nitrate, ammonium, and phosphate in a sandy soil. Chemosphere.

[15] Yu O-Y, Raichle B, Sink S (2013). Impact of biochar on the water holding capacity of loamy sand soil. Int J Energy Environ Eng.

[16] Cayuela ML, Jeffery S, van Zwieten L (2015). The molar H:Corg ratio of biochar is a key factor in mitigating N_2_O emissions from soil. Agr Ecosyst Environ.

[17] Pfab H (2011). N_2_O fluxes from a Haplic Luvisol under intensive production of lettuce and cauliflower as affected by different N-fertilization strategies. J Plant Nutr Soil Sci.

[18] Zona D (2011). Impact of extreme precipitation and water table change on N_2_O fluxes in a bio-energy poplar plantation. Biogeosciences Discuss.

[19] Philippot L, Hallin S, Schloter M (2007). Ecology of denitrifying prokaryotes in agricultural soil. Adv Agron.

[20] Braker G, Conrad R (2011). Diversity, structure, and size of N_2_O-producing microbial communities in soils - what matters for their functioning?. Adv Appl Microbiol.

[21] Richardson D, Felgate H, Watmough N, Thomson A, Baggs E (2009). Mitigating release of the potent greenhouse gas N_2_O from the nitrogen cycle - could enzymic regulation hold the key?. Trends Biotechnol.

[22] Bakken LR, Bergaust L, Liu BB, Frostegard A (2012). Regulation of denitrification at the cellular level: a clue to the understanding of N_2_O emissions from soils. Philos Trans R Soc, B.

[23] Philippot L, Andert J, Jones CM, Bru D, Hallin S (2011). Importance of denitrifiers lacking the genes encoding the nitrous oxide reductase for N_2_O emissions from soil. Global Change Biol.

[24] Mckenney DJ (1994). Kinetics of Denitrification by *Pseudomonas fluorescens*: Oxygen Effects. Soil Biol Biochem.

[25] Zumft, W. G. & Körner, H. In *Biology of the Nitrogen Cycle* (eds H. Bothe, S. J. Ferguson & W. E. Newton) Ch. 5, 67–82 (Elsevier, 2007).

[26] Jones CM, Graf DR, Bru D, Philippot L, Hallin S (2013). The unaccounted yet abundant nitrous oxide-reducing microbial community: a potential nitrous oxide sink. Isme J.

[27] Sanford RA (2012). Unexpected nondenitrifier nitrous oxide reductase gene diversity and abundance in soils. P Natl Acad Sci USA.

[28] Orellana LH (2014). Detecting Nitrous Oxide Reductase (*nosZ*) Genes in Soil Metagenomes: Method Development and Implications for the Nitrogen Cycle. Mbio.

[29] Jones CM (2014). Recently identified microbial guild mediates soil N_2_O sink capacity. Nat Clim Change.

[30] Anderson CR (2011). Biochar induced soil microbial community change: Implications for biogeochemical cycling of carbon, nitrogen and phosphorus. Pedobiologia.

[31] Xu HJ (2014). Biochar Impacts Soil Microbial Community Composition and Nitrogen Cycling in an Acidic Soil Planted with Rape. Environ Sci Technol.

[32] Harter J (2014). Linking N_2_O emissions from biochar-amended soil to the structure and function of the N-cycling microbial community. Isme J.

[33] Harter J (2016). Soil biochar amendment shapes the composition of N_2_O-reducing microbial communities. Sci Total Environ.

[34] Harter J (2016). Gas entrapment and microbial N_2_O reduction reduce N_2_O emissions from a biochar-amended sandy clay loam soil. Sci Rep.

[35] Chen JH (2015). Consistent increase in abundance and diversity but variable change in community composition of bacteria in topsoil of rice paddy under short term biochar treatment across three sites from South China. Appl Soil Ecol.

[36] Anderson CR, Hamonts K, Clough TJ, Condron LM (2014). Biochar does not affect soil N-transformations or microbial community structure under ruminant urine patches but does alter relative proportions of nitrogen cycling bacteria. Agr Ecosyst Environ.

[37] Van Zwieten L (2014). An incubation study investigating the mechanisms that impact N_2_O flux from soil following biochar application. Agr Ecosyst Environ.

[38] Ducey TF, Ippolito JA, Cantrell KB, Novak JM, Lentz RD (2013). Addition of activated switchgrass biochar to an aridic subsoil increases microbial nitrogen cycling gene abundances. Appl Soil Ecol.

[39] Lane, D. J. In *Nucleic acid techniques in bacterial systematics* (eds E. Stackebrandt & M. Godfellow) Ch. 6, 115–175 (Wiley, 1991).

[40] Wilson KH, Blitchington RB, Greene RC (1990). Amplification of Bacterial 16S Ribosomal DNA with Polymerase Chain Reaction. J Clin Microbiol.

[41] Throback IN, Enwall K, Jarvis A, Hallin S (2004). Reassessing PCR primers targeting *nirS*, *nirK* and *nosZ* genes for community surveys of denitrifying bacteria with DGGE. Fems Microbiol Ecol.

[42] Henry S, Bru D, Stres B, Hallet S, Philippot L (2006). Quantitative detection of the *nosZ* gene, encoding nitrous oxide reductase, and comparison of the abundances of 16S rRNA, *narG*, *nirK*, and *nosZ* genes in soils. Appl Environ Microb.

[43] Martin M (2011). Cutadapt removes adapter sequences from high-throughput sequencing reads. EMBnet. journal.

[44] Edgar RC (2010). Search and clustering orders of magnitude faster than BLAST. Bioinformatics.

[45] Edgar RC, Flyvbjerg H (2015). Error filtering, pair assembly and error correction for next-generation sequencing reads. Bioinformatics.

[46] Edgar RC, Haas BJ, Clemente JC, Quince C, Knight R (2011). UCHIME improves sensitivity and speed of chimera detection. Bioinformatics.

[47] Buchfink B, Xie C, Huson DH (2015). Fast and sensitive protein alignment using DIAMOND. Nat Methods.

[48] Huson DH, Mitra S, Ruscheweyh HJ, Weber N, Schuster SC (2011). Integrative analysis of environmental sequences using MEGAN4. Genome Res.

[49] Huson DH (2016). MEGAN Community Edition - Interactive Exploration and Analysis of Large-Scale Microbiome Sequencing Data. PLoS Comput Biol.

[50] Huson DH, Auch AF, Qi J, Schuster SC (2007). MEGAN analysis of metagenomic data. Genome Res.

[51] Asnicar F, Weingart G, Tickle TL, Huttenhower C, Segata N (2015). Compact graphical representation of phylogenetic data and metadata with GraPhlAn. Peerj.

[52] Oksanen, J. *et al*. vegan: Community Ecology Package. *R package version 2*.*4*-*2* (2017).

[53] Palmer K, Horn MA (2015). Denitrification Activity of a Remarkably Diverse Fen Denitrifier Community in Finnish Lapland Is N-Oxide Limited. PloS one.

[54] Bremer C (2007). Impact of plant functional group, plant species, and sampling time on the composition of *nirK*-Type denitrifier communities in soil. Appl Environ Microb.

[55] Henry S (2008). Disentangling the rhizosphere effect on nitrate reducers and denitrifiers: insight into the role of root exudates. Environ Microbiol.

[56] Palmer K, Biasi C, Horn MA (2012). Contrasting denitrifier communities relate to contrasting N_2_O emission patterns from acidic peat soils in arctic tundra. Isme J.

[57] Hu L, Cao L, Zhang R (2014). Bacterial and fungal taxon changes in soil microbial community composition induced by short-term biochar amendment in red oxidized loam soil. World J Microbiol Biotechnol.

[58] Graber ER (2010). Biochar impact on development and productivity of pepper and tomato grown in fertigated soilless media. Plant Soil.

[59] Wang CY (2015). The chemical composition of native organic matter influences the response of bacterial community to input of biochar and fresh plant material. Plant Soil.

[60] Thies, J. E. & Rillig, M. C. In *Biochar for environmental management science and technology* (eds J. Lehmann & S. Joseph) Ch. 6 (Earthscan, 2009).

[61] Elboutahiri N, Thami-Alami I, Udupa SM (2010). Phenotypic and genetic diversity in *Sinorhizobium meliloti* and *S*. *medicae* from drought and salt affected regions of Morocco. BMC Microbiol.

[62] Buendiaclaveria AM, Rodrigueznavarro DN, Santamarialinaza C, Ruizsainz JE, Tempranovera F (1994). Evaluation of the Symbiotic Properties of *Rhizobium fredii* in European Soils. Syst Appl Microbiol.

[63] Delamuta JRM (2013). Polyphasic evidence supporting the reclassification of *Bradyrhizobium* j*aponicum* group la strains as *Bradyrhizobium diazoefficiens* sp nov. Int J Syst Evol Micr.

[64] Krause A (2006). Complete genome of the mutualistic, N_2_-fixing grass endophyte *Azoarcus* sp strain BH72. Nat Biotechnol.

[65] Markowitz VM (2014). IMG 4 version of the integrated microbial genomes comparative analysis system. Nucleic Acids Res.

[66] Delgado, M. J., Casella, S. & Bedmar, E. J. In *Biology of the Nitrogen Cycle* (eds H. Bothe, S. J. Ferguson & W. E. Newton) Ch. 6, 83–91 (Elsevier, 2007).

[67] Tago K, Ishii S, Nishizawa T, Otsuka S, Senoo K (2011). Phylogenetic and Functional Diversity of Denitrifying Bacteria Isolated from Various Rice Paddy and Rice-Soybean Rotation Fields. Microbes Environ.

[68] Bueno E (2015). Anoxic growth of *Ensifer meliloti* 1021 by N_2_O-reduction, a potential mitigation strategy. Front Microbiol.

[69] Sanchez C, Itakura M, Mitsui H, Minamisawa K (2013). Linked Expressions of *nap* and *nos* Genes in a *Bradyrhizobium japonicum* Mutant with Increased N_2_O Reductase Activity. Appl Environ Microb.

[70] Zumft WG, Kroneck PMH (2007). Respiratory transformation of nitrous oxide (N_2_O) to dinitrogen by Bacteria and Archaea. Adv Microb Physiol.

[71] Graf DRH, Jones CM, Hallin S (2014). Intergenomic Comparisons Highlight Modularity of the Denitrification Pathway and Underpin the Importance of Community Structure for N_2_O Emissions. PloS one.

[72] Matocha, C. J., Dhakal, P. & Pyzola, S. M. In *Advances in Agronomy*, *Volume 115* (ed. D. L. Sparks) Ch. 4, 181–214 (Academic Press, 2012).

[73] Lehmann J (2011). Biochar effects on soil biota - A review. Soil Biol Biochem.

[74] Khodadad CLM, Zimmerman AR, Green SJ, Uthandi S, Foster JS (2011). Taxa-specific changes in soil microbial community composition induced by pyrogenic carbon amendments. Soil Biol Biochem.

[75] Kappler A (2014). Biochar as an Electron Shuttle between Bacteria and Fe(III) Minerals. Environmental Science & Technology Letters.

[76] Hagemann, N., Harter, J. & Behrens, S. In *Biochar Application*: *Essential Soil Microbial Ecology* (eds T. Komang Ralebitso-Senior & C. Orr) Ch. 8, 163–198 (Elsevier, 2016).

[77] Albareda M, Rodriguez-Navarro DN, Temprano FJ (2009). Use of *Sinorhizobium* (*Ensifer*) *fredii* for soybean inoculants in South Spain. Eur J Agron.

[78] Latha S, Mahadevan A (1997). Role of rhizobia in the degradation of aromatic substances. World J Microbiol Biotechnol.

