# Supplementary material for: Soil biochar amendment affects the diversity of nosZ transcripts: Implications for N2O formation
Source: Sci Rep. 2017 Jun 13;7:3338. doi: 10.1038/s41598-017-03282-y (PMC5469825; doi:10.1038/s41598-017-03282-y)
Supplement: Supplementary file 1 — Supplementary Information [file 41598_2017_3282_MOESM1_ESM.pdf]

Soil biochar amendment affects the diversity of *nosZ* transcripts: Implications for N_2O formation

Johannes Harter [1], Mohamed El-Hadidi [2], Daniel H. Huson [2], Andreas Kappler [1], Sebastian Behrens [4,5,*]

[1] Geomicrobiology & Microbial Ecology, Center for Applied Geosciences, University of Tuebingen, Tuebingen, Germany
[2] Algorithms in Bioinformatics, Center for Bioinformatics, University of Tuebingen, Tuebingen, Germany
[3] Bioinformatics, Center for Informatics Science (CIS), Nile University, Giza, Egypt
[4] Department of Civil, Environmental, and Geo-Engineering, University of Minnesota, Minneapolis, MN, USA
[5] BioTechnology Institute, University of Minnesota, St. Paul, MN, USA

Supplementary information

* Corresponding author (Tel.: +1-612-626-8225 and +1-612-624-8454. Email: sbehrens@umn.edu).

Table S1: Reaction mixtures and thermal profiles of the PCRs used to amplify *nirK*, typical *nosZ*, and atypical *nosZ* transcripts.

**target gene**	**reaction mixture**	**volume (µl)**	**thermal profile**
nirK	FastStart HiFi Polymerase (5 U/µl)	0.25	94°C – 5 min<br>(1 cycle)<br>94°C – 30 s<br>56°C – 45 s<br>72°C - 60 s<br>(40 cycles)<br>72°C - 8 min<br>(1 cycle)
	FastStart 10 x Buffer (with 18 mM $MgCl_2$)	2.5	
	dNTP mix (10 mM each)	0.5	
	F1aCu_OA (10 µM)	1	
	R3Cu_OA (10 µM)	1	
	DMSO	0.5	
	BSA	0.25	
	PCR water	16	
	cDNA sample	3	
typical *nosZ*	FastStart HiFi Polymerase (5 U/µl)	0.25	94°C – 5 min<br>(1 cycle)<br>94°C – 30 s<br>56°C – 45 s<br>72°C - 60 s<br>(40 cycles)<br>72°C - 8 min<br>(1 cycle)
	FastStart 10 x Buffer (without $MgCl_2$)	2.5	
	$MgCl_2$ (25 mM)	3.5	
	dNTP mix (10 mM each)	0.5	
	nosZ2F_OA (10 µM)	2	
	nosZ2R_OA (10 µM)	2	
	DMSO	1	
	BSA	0.25	
	PCR water	10	
	cDNA sample	3	
atypical *nosZ*	FastStart HiFi Polymerase (5 U/µl)	0.25	94°C – 8 min<br>(1 cycle)<br>94°C – 45 s<br>56°C – 45 s<br>72°C - 60 s<br>(40 cycles)<br>72°C - 8 min<br>(1 cycle)
	FastStart 10 x Buffer (without $MgCl_2$)	2.5	
	$MgCl_2$ (25 mM)	3.5	
	dNTP mix (10 mM each)	0.5	
	nosZ-II-F_OA (10 µM)	2	
	nosZ-II-R_OA (10 µM)	2	
	DMSO	2	
	BSA	0.5	
	PCR water	8.75	
	cDNA sample	3	

Primers consist of target-specific primers (F1aCu/R3Cu, nosZ2F/nosZ2R, and nosZ-II-F/nosZ-II-R) with overhang adapters.
F1aCu_OA: 5'-TCGTCGGCAGCGTCAGATGTGTATAAGAGACAGATCATGGTSCTGCCGCG-3'
R3Cu_OA: 5'-GTCTCGTGGGCTCGGAGATGTGTATAAGAGACAGGCCTCGATCAGRTTGTGGTT-3'
nosZ2F_OA: 5'-TCGTCGGCAGCGTCAGATGTGTATAAGAGACAGCGCRACGGCAASAAGGTSMSSGT-3'
nosZ2R_OA: 5'-GTCTCGTGGGCTCGGAGATGTGTATAAGAGACAGCAKRTGCAKSGCRTGGCAGAA-3'
nosZ-II-F_OA: 5'-TCGTCGGCAGCGTCAGATGTGTATAAGAGACAGCTNGGNCCNYTKCAYAC-3'
nosZ-II-R_OA: 5'-GTCTCGTGGGCTCGGAGATGTGTATAAGAGACAGGCNGARCARAANTCBGTRC-3'

Table S2: Relative sequence abundances of all *nirK*-expressing microbial species.

***nirK*-expressing species**	**rel. seq. abundance [%]**
Mesorhizobium ciceri	47.0
Rhodopseudomonas palustris	13.3
Mesorhizobium opportunistum	6.12
Mesorhizobium australicum	4.02
Chelativorans sp. BNC1	2.05
Bradyrhizobium BTAi1	0.48
Ensifer fredii	0.44
Ochrobactrum anthropi	0.39
Ensifer medicae	0.35
Bradyrhizobium oligotrophicum	0.08
Burkholderia multivorans	0.06
Nitrosomonas sp. AL212	0.06
Hyphomicrobium nitrativorans	0.03
Shewanella loihica	0.03
Bradyrhizobium ORS-278	0.02
Rhizobium etli	0.01
Achromobacter xylosoxidans	<0.01
Phaeobacter gallaeciensis	<0.01
Pseudomonas mendocina	<0.01
Rhodobacter sphaeroides	<0.01

Table S3: Relative sequence abundances of all typical *nosZ*-expressing microbial species.

**typical *nosZ*-expressing species**	**rel. seq. abundance [%]**
Oligotropha carboxidovorans	31.8
Bradyrhizobium diazoefficiens	15.1
Hyphomicrobium nitrativorans	12.4
Ensifer meliloti	10.6
Azoarcus sp. KH32C	6.57
Ensifer fredii	4.68
Polymorphum gilvum	2.45
Azoarcus sp. BH72	1.24
Rhodobacter sphaeroides	0.86
Bradyrhizobium sp. BTAi1	0.81
Rubrivivax gelatinosus	0.76
Rhodospirillum centenum	0.41
Pseudomonas stutzeri	0.39
Thiobacillus denitrificans	0.39
Azospirillum lipoferum	0.21
Pseudomonas brassicacearum	0.17
Pseudomonas fluorescens	0.17
Anaeromyxobacter dehalogenans	0.16
Dinoroseobacter shibae	0.16
Ralstonia pickettii	0.15
Pseudomonas aeruginosa	0.14
Ochrobactrum anthropi	0.10
Gammaproteobacterium HdN1	0.09
Pseudogulbenkiania sp. NH8B	0.07
Rhodoferax ferrireducens	0.07
Paracoccus denitrificans	0.06
Leptothrix cholodnii	0.04
Hyphomicrobium denitrificans	0.03
Rhodopseudomonas palustris	0.03
Roseobacter denitrificans	0.03
Bordetella petrii	0.02
Anaeromyxobacter sp. Fw109-5	0.01
Aromatoleum aromaticum	0.01
Methylobacterium sp. 4-46	0.01
Ralstonia solanacearum	0.01
Alicycliphilus denitrificans	<0.01
Bradyrhizobium oligotrophicum	<0.01
Burkholderia multivorans	<0.01
Janthinobacterium sp. Marseille	<0.01
Legionella pneumophila	<0.01
Rhodospirillum photometricum	<0.01
Sphingomonas wittichii	<0.01
Stigmatella aurantiaca	<0.01

Table S4: Relative sequence abundances of all atypical *nosZ*-expressing microbial species.

atypical *nosZ*-expressing species	rel. seq. abundance [%]
Melioribacter roseus	35.3
Caldilinea aerophila	12.6
Taylorella asinigenitalis	7.08
Burkholderia multivorans	4.43
Owenweeksia hongkongensis	3.75
Pedobacter saltans	3.01
Belliella baltica	2.72
Mesorhizobium ciceri	2.44
Clostridium botulinum	2.23
Gemmatimonas aurantiaca	1.92
Opitutus terrae	1.66
Thermomicrobium roseum	1.62
Ignavibacterium album	1.50
Runella slithyformis	1.36
Flavobacteriaceae bacterium 3519-10	1.17
Legionella pneumophila	0.80
Azospirillum lipoferum	0.77
Carnobacterium maltaromaticum	0.77
Lactobacillus crispatus	0.71
Rhodospirillum photometricum	0.63
Rhodopseudomonas palustris	0.59
Chelativorans sp. BNC1	0.46
Niastella koreensis	0.44
Clostridium tetani	0.42
Heliobacterium modesticaldum	0.36
Corynebacterium efficiens	0.34
Persephonella marina	0.28
Enterococcus mundtii	0.27
Shewanella piezotolerans	0.26
Blattabacterium sp. (Nauphoeta cinerea)	0.24
Anaeromyxobacter sp. Fw109-5	0.20
Mesorhizobium australicum	0.20
Nocardia cyriacigeorgica	0.18
Mesorhizobium opportunistum	0.16
Streptococcus dysgalactiae	0.14
Bacillus subtilis	0.11
Solitalea canadensis	0.07
Ensifer fredii	0.06
Hydrogenobacter thermophilus	0.05
Bradyrhizobium oligotrophicum	0.04
Flavobacterium columnare	0.04
Desulfotomaculum ruminis	0.03
Mycobacterium rhodesiae	0.03
Dyadobacter fermentans	0.02
Ensifer medicae	0.02
Helicobacter felis	0.02
Rhodothermus marinus	0.02
Azoarcus sp. KH32C	0.01
Bacillus coagulans	0.01
Bradyrhizobium sp. ORS 278	0.01
Haliscomenobacter hydrossis	0.01
Muricauda ruestringensis	0.01
Ochrobactrum anthropi	0.01
Riemerella anatipestifer	0.01
Sphaerobacter thermophilus	0.01
Sulfuricella denitrificans	0.01
